# Kidney health for everyone everywhere – from prevention to detection and equitable access to care

**DOI:** 10.1590/1414-431X20209614

**Published:** 2020-03-09

**Authors:** P. Kam-Tao Li, G. Garcia-Garcia, Siu-Fai Lui, S. Andreoli, W. Wing-Shing Fung, A. Hradsky, L. Kumaraswami, V. Liakopoulos, Z. Rakhimova, G. Saadi, L. Strani, I. Ulasi, K. Kalantar-Zadeh

**Affiliations:** 1 Department of Medicine and Therapeutics, Carol & Richard Yu PD Research Centre, Prince of Wales Hospital, Chinese University of Hong Kong, Hong Kong; 2 Nephrology Service, Hospital Civil de Guadalajara Fray Antonio Alcalde, University of Guadalajara Health Sciences Center, Guadalajara, Mexico; 3 Division of Health System, Policy and Management, Jockey Club School of Public Health and Primary Care, The Chinese University of Hong Kong, Hong Kong; 4 James Whitcomb Riley Hospital for Children, Indiana University School of Medicine, Indianapolis, IN, USA; 5 World Kidney Day Office, Brussels, Belgium; 6 Tanker Foundation, Chennai, India; 7 Division of Nephrology and Hypertension, 1st Department of Internal Medicine, AHEPA Hospital, Aristotle University of Thessaloniki, Thessaloniki, Greece; 8 Nephrology Unit, Department of Internal Medicine, Faculty of Medicine, Cairo University, Giza, Egypt; 9 Renal Unit, Department of Medicine, College of Medicine, University of Nigeria, Ituku-Ozalla, Enugu, Nigeria; 10 Division of Nephrology and Hypertension and Kidney Transplantation, University of California Irvine School of Medicine, Orange, CA, USA

**Keywords:** Kidney diseases, Prevention, Detection, Awareness

## Abstract

The global burden of chronic kidney disease (CKD) is rapidly increasing with a projection of becoming the 5th most common cause of years of life lost globally by 2040. CKD is a major cause of catastrophic health expenditure. The costs of dialysis and transplantation consume up to 3% of the annual healthcare budget in high-income countries. However, the onset and progression of CKD is often preventable. In 2020, the World Kidney Day campaign highlights the importance of preventive interventions – be it primary, secondary, or tertiary. This article focuses on outlining and analyzing measures that can be implemented in every country to promote and advance CKD prevention. Primary prevention of kidney disease should focus on the modification of risk factors and addressing structural abnormalities of the kidney and urinary tracts, as well as exposure to environmental risk factors and nephrotoxins. In persons with pre-existing kidney disease, secondary prevention, including blood pressure optimization and glycemic control, should be the main goal of education and clinical interventions. In patients with advanced CKD, management of co-morbidities such as uremia and cardiovascular disease is a highly recommended preventative intervention to avoid or delay dialysis or kidney transplantation. Political efforts are needed to proliferate the preventive approach. While national policies and strategies for non-communicable diseases might be present in a country, specific policies directed toward education and awareness about CKD screening, management, and treatment are often lacking. Hence, there is an urgent need to increase the awareness of preventive measures throughout populations, professionals, and policy makers.

## Introduction

Around 850 million people currently are affected by different types of kidney disorders (1). Up to one in ten adults worldwide has chronic kidney disease (CKD), which is invariably irreversible and mostly progressive. The global burden of CKD is increasing, and CKD is projected to become the 5th most common cause of years of life lost globally by 2040 (2). If CKD remains uncontrolled and if the affected person survives the ravages of cardiovascular and other complications of the disease, CKD progresses to end-stage renal disease (ESRD), where life cannot be sustained without dialysis therapy or kidney transplantation. Hence, CKD is a major cause of catastrophic health expenditure (3). The costs of dialysis and transplantation consume 2–3% of the annual health-care budget in high-income countries, spent on less than 0.03% of the total population of these countries (4 [Bibr B05] [Bibr B06]).

Importantly, however, kidney disease can be prevented and progression to ESRD can be delayed with appropriate access to basic diagnostics and early treatment including life style modifications and nutritional interventions (4–8). Despite this, access to effective and sustainable kidney care remains highly inequitable across the world, and kidney disease remains a low health priority in many countries. Kidney disease is missing from the international agenda for global health. Notably absent from the impact indicators for the Sustainable Development Goal 3, Target 3.4 (by 2030, reduce by one third premature mortality from non-communicable diseases (NCDs) through prevention and treatment and promote mental health and well-being) and the latest iteration of the Untied Nation (UN) Political Declaration on NCDs, kidney diseases urgently need to be given political attention, priority, and consideration (9). Current global political commitments on NCDs focus largely on four main diseases: cardiovascular disease (CVD), cancer, diabetes, and chronic respiratory diseases. Yet, it is estimated that 55% of the global NCD burden is attributed to diseases outside of this group (10). Furthermore, kidney disease frequently co-exists with the ‘big' four NCDs, which leads to worse health outcomes. CKD is a major risk factor for heart disease and cardiac death, as well as for infections such as tuberculosis, and is a major complication of other preventable and treatable conditions including diabetes, hypertension, HIV, and hepatitis (4–7). As the Sustainable Development Goals and Universal Health Coverage agendas progress and provide a platform for raising awareness of NCD health care and monitoring needs, targeted action on kidney disease prevention should become integral to the global policy response (1). The global kidney health community calls for the recognition of kidney disease and effective identification and management of its risk factors as a key contributor to the global NCD burden and the implementation of an integrated and people's centered approach to care.

## Definition and classification of CKD prevention

**Figure 1.**
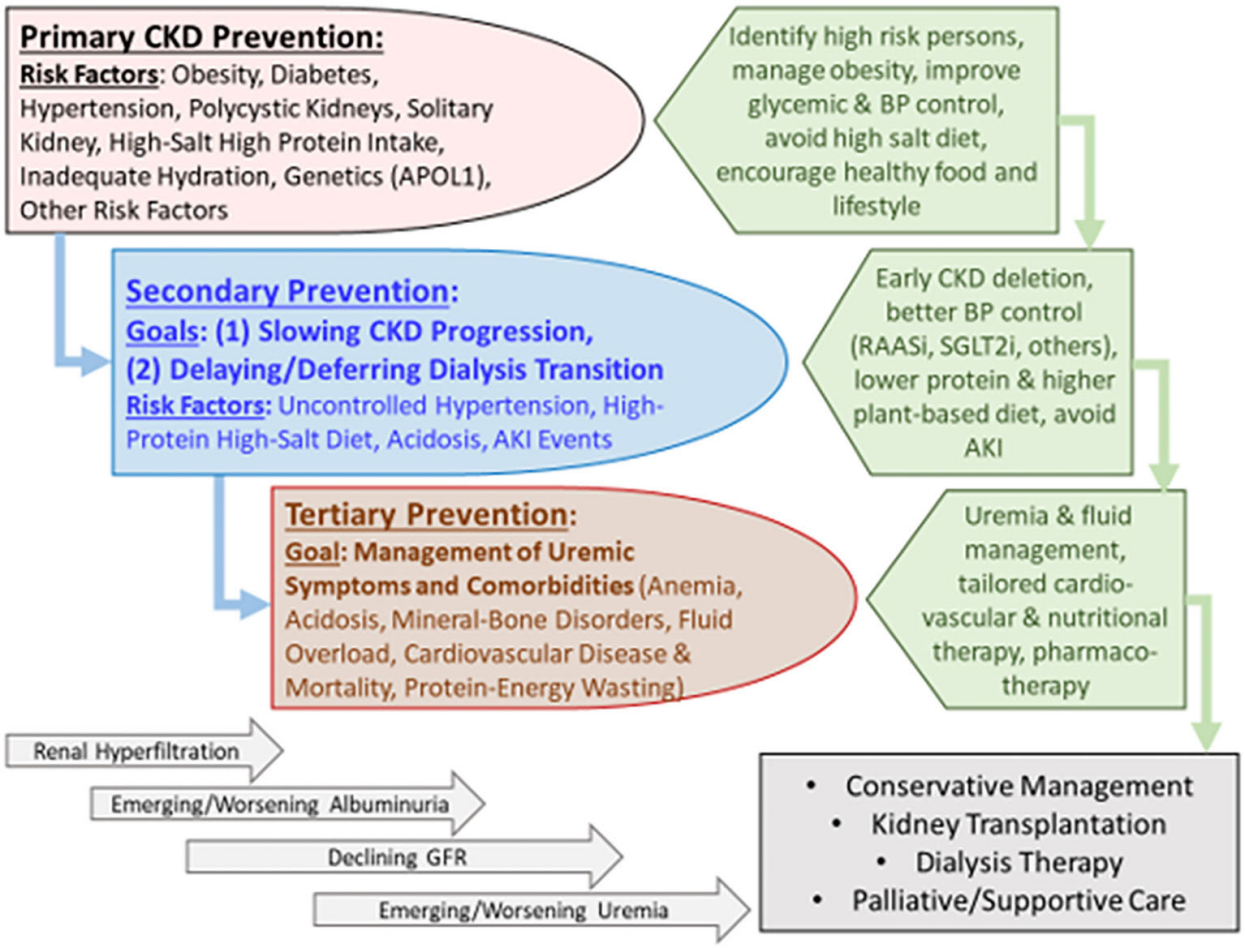
Overview of the preventive measures in chronic kidney disease (CKD) to highlight the similarities and distinctions pertaining to primary, secondary, and tertiary preventive measures and their intended goals. AKI: acute kidney injury; GFR: glomerular filtration rate; BP: blood pressure; RAASi: renin-angiotensin-aldosterone system inhibitors; SGLT2i: sodium-glucose cotransporter-2 inhibitors.

According to the expert definitions including the Center for Disease Control and Prevention (11), the term “prevention” refers to activities that are typically categorized by the following three definitions: 1) Primary Prevention implies intervening before health effects occur in an effort to prevent the onset of illness or injury before the disease process begins; 2) Secondary Prevention suggests preventive measures that lead to early diagnosis and prompt treatment of a disease to prevent more severe problems and includes screening to identify diseases in the earliest stages; and 3) Tertiary Prevention indicates managing disease after it is well established in order to control disease progression and the emergence of more severe complications, which is often by means of targeted measures such as pharmacotherapy, rehabilitation, and screening for and management of complications. These definitions have important bearing on the prevention and management of CKD, and accurate identification of risk factors that cause CKD or lead to faster progression to renal failure as shown in Figure 1 are relevant in health policy decisions and health education and awareness related to CKD (12).

## Primary prevention of CKD

The incidence (new cases) and prevalence (cumulative pre-existing cases) of CKD have been rising worldwide (13). This primary level of prevention requires awareness of modifiable CKD risk factors and efforts to focus healthcare resources on those patients who are at the highest risk of developing new onset or *de novo* CKD.

Measures to achieve effective primary prevention should focus on the two leading risk factors for CKD including diabetes mellitus and hypertension. Evidence suggests that an initial mechanism of injury is renal hyperfiltration with seemingly elevated glomerular filtration rate (GFR), above normal ranges. This is often the result of glomerular hypertension that is often seen in patients with obesity or diabetes mellitus, but it can also occur after a high dietary protein intake (8). Other CKD risk factors include polycystic kidneys or other congenital or acquired structural anomalies of the kidney and urinary tracts, primary glomerulonephritis, exposure to nephrotoxic substances or medications (such as nonsteroidal anti-inflammatory drugs), having one single kidney, e.g., solitary kidney after cancer nephrectomy, high dietary salt intake, inadequate hydration with recurrent volume depletion, heat stress, exposure to pesticides and heavy metals (as has been speculated as the main cause of Mesoamerican nephropathy), and possibly high protein intake in those at higher risk of CKD (8). Among non-modifiable risk factors are advancing age and genetic factors such as apolipoprotein 1 (APOL1) gene that is mostly encountered in those with sub-Saharan African ethnicity, especially among African Americans. Certain disease states may cause *de novo* CKD such as cardiovascular and atheroembolic diseases (also known as secondary cardiorenal syndrome) and liver diseases (hepatorenal syndrome). Table 1 shows some of the risk factors of CKD.

**Table 1.** Risk factors for *de novo* chronic kidney disease (CKD) and pre-existing CKD progression.

Risk Factor^*^	Contribution to *de novo* CKD	Contribution to CKD progression
Diabetes mellitus	∼50% of all CKDs	
Hypertension	∼25% of all CKDs	
Obesity	10–20%	
Age	Seen with advancing age, especially in the setting of comorbid conditions	Some suggest that older CKD patients may have slower progression
Race, genetics, and other hereditary factors APOL1 gene Hereditary nephritis (Alport's)	Common among those with African American ancestors	
Acute glomerulonephritis (GN) Post-infectious GN Rapidly progressive GN	<10%	Recurrent GN or exacerbation of proteinuria
Polycystic kidney disorders	<10%, family history of cystic kidney disorders	
Acute kidney injury (AKI) Acute tubular necrosis (ATN) Acute interstitial nephritis (AIN)	Repeated AKI bouts can cause CKD	Repeated AKI bouts can accelerate CKD progression
Autoimmune disorders Lupus erythematosus Other connective tissue disorders		
Pharmacologic Medications causing interstitial nephritides (NSAIDs, CNI, chemotherapy, PPI, etc) or ATN (aminoglycosides) Herbs and herbal medication	Variable, e.g., in Taiwan, Chinese herb nephropathy may be an important contributor	
Environmental Heavy metal exposure	Rare	
Acquired or congenital solitary kidney Cancer, donor or traumatic nephrectomy Congenital solitary kidney, unilateral atrophic kidney		
Acquired urinary tract disorders & obstructive nephropathy	Benign prostate hyperplasia in menGynecological cancers in women	
Congenital anomalies of the kidney and urinary tract	Mostly in children and young adults	
Inadequate fluid intake Mesoamerican nephropathy Others	Unknown risk, but high prevalence is suspected in Central America	Whereas in earlier CKD stages adequate hydration is important to avoid pre-renal AKI bouts, higher fluid intake in more advanced CKD may increase the risk of hyponatremia
High protein intake	Unknown risk, recent data suggest higher CKD risk or faster CKD progression with high protein diet, in particular from animal sources	Higher protein intake can accelerate the rate of CKD progression
Cardiovascular diseases (cardiorenal)	Ischemic nephropathy	
Liver disease (hepatorenal)	Non-alcoholic steatohepatitis, viral hepatitis	

^*^ Many of these risk factors contribute to both *de novo* CKD and its faster progression and, hence, are relevant to both primary and secondary prevention.

Among measures to prevent emergence of *de novo* CKD are screening efforts to identify and manage persons at high risk of CKD, especially those with diabetes mellitus and hypertension. Hence, targeting primordial risk factors of these two conditions including metabolic syndrome and overnutrition is relevant to primary CKD prevention as is correcting obesity (14). Promoting healthier lifestyle is an important means to that end including physical activity and healthier diet. The latter should be based on more plant-based foods with less meat, less sodium intake, more complex carbohydrates with higher fiber intake, and less saturated fat. In those with hypertension and diabetes, optimizing blood pressure and glycemic control has shown to be effective in preventing diabetic and hypertensive nephropathies. A recent expert panel suggested that persons with solitary kidney should avoid high protein intake above 1 g/kg body weight per day (15). Obesity should be avoided, and weight reduction strategies should be considered (14).

## Secondary prevention in CKD

Evidence suggests that among those with CKD, the vast majority have early-stage of the disease. i.e., CKD stages 1 and 2 with microalbuminuria (30 to 300 mg/day) or CKD stage 3B (eGFR between 45 to 60 mL·min^-1^·(1.73 m^2^)^-1^) (16). In these persons with preexisting disease, the “secondary prevention” of CKD has the highest priority. For these earlier stages of CKD, the main goal of kidney health education and clinical interventions is how to slow disease progression. Uncontrolled or poorly controlled hypertension is one of the most established risk factors for faster CKD progression. The underlying pathophysiology of faster CKD progression relates to ongoing damage to the kidney structure and loss of nephrons with worsening interstitial fibrosis as occurs with sustained hypertension.

The cornerstone of pharmacotherapy in secondary prevention is the use of angiotensin pathway modulators, also known as renin-angiotensin-aldosterone system inhibitors (RAASi). These drugs reduce both systemic blood pressure and intraglomerular pressure by opening efferent arterioles of the glomeruli, hence, leading to longevity of the remaining nephrons. Low protein diet appears to have a synergistic effect on RAASi therapy (17). In terms of the potential effect of controlling glycemic status and correcting obesity on the rate of CKD progression, there are mixed data. However, recent data suggest that a new class of anti-diabetic medications known as sodium-glucose cotransporter-2 inhibitors (SGLT2i) can slow CKD progression, but this effect may not be related to the glycemic modulation of the medication. Whereas acute kidney injury (AKI) may or may not cause *de novo* CKD, AKI events that are superimposed on preexisting CKD may accelerate disease progression (18). A relatively recent case of successful secondary prevention that highlights the significance of implementing preventive strategies in CKD is the use of a vasopressin type-2-receptor antagonists in adult polycystic kidney disease (19).

## Tertiary prevention in CKD

In patients with advanced CKD, management of uremia and related comorbid conditions such as anemia, mineral and bone disorders, and cardiovascular disease is of high priority, so that these patients can achieve the highest longevity. These measures can be collectively referred to as “tertiary prevention” of CKD. In these individuals, cardiovascular disease burden is exceptionally high, especially if they have underlying diabetes or hypertension, while they often do not follow other traditional profiles of cardiovascular risk such as obesity or hyperlipidemia. Indeed, in these patients, a so-called “reverse epidemiology” exists, in that hyperlipidemia and obesity appear to be protective at this advanced stage of CKD. This could be due to the overshadowing impact of the “protein-energy wasting” (PEW) that happens more frequently with worsening uremia and which is associated with weight loss and poor outcomes including cardiovascular disease and death. Whereas many of these patients, if they survive the ravages of PEW and cardiovascular disease, will eventually receive renal replacement therapy in the form of dialysis therapy or kidney transplantation, a new trend is emerging to maintain them longer without dialysis by implementing conservative management of CKD. However, in some with additional comorbidities such as metastatic cancers, palliative measures with supportive care can be considered.

## Approaches to identification of CKD

The lack of awareness of CKD around the world is one of the reasons for late presentation of CKD in both developed and developing economies (20–22). The overall CKD awareness among the general population and even high cardiovascular risk groups across 12 low-income and middle-income countries (LMIC) was less than 10% (22).

Given its asymptomatic nature, screening of CKD plays an important role in early detection. Consensus and positional statements have been published by the International Society of Nephrology (ISN) (23), the National Kidney Foundation (24), the Kidney Disease Improving Global Outcomes (25), the NICE Guidelines (26), and the Asian Forum for CKD Initiatives (27). There was a lack of trials to evaluate screening and monitoring of CKD (28). Currently, most will promote a targeted screening approach to early detection of CKD. Some of the major groups at risk for targeted screening include: patients with diabetes, hypertension, those with family history of CKD, individuals receiving potentially nephrotoxic drugs, herbs or substances or taking indigenous medicine, patients with past history of acute kidney injury, and individuals older than 65 years of age (27,29). CKD can be detected with 2 simple tests: a urine test for the detection of proteinuria and a blood test to estimate the GFR (24,27).

Given that currently population screening for CKD is not recommended and it was claimed that it might add unintended harm to the general population being screened (28), there is no specialty society or preventive services group that recommends general screening (30). LMIC are ill-equipped to deal with the devastating consequences of CKD, particularly the late stages of the disease. There are suggestions that screening should primarily include high-risk individuals, but also extend to those with suboptimal levels of risk, e.g., prediabetes and prehypertension (31).

## Cost-effectiveness of early detection programs

Universal screening of the general population would be time-consuming, expensive, and has been shown to be not cost-effective. Unless selectively directed towards high-risk groups, such as the case of CKD in disadvantaged populations (32), according to a cost-effectiveness analysis using a Markov decision analytic model, population-based dipstick screening for proteinuria has an unfavorable cost effectiveness ratio (33). A more recent Korean study confirmed that their National Health Screening Program for CKD is more cost-effective for patients with diabetes or hypertension than the general population (34). From an economic perspective, screening CKD by detection of proteinuria was shown to be cost-effective in patients with hypertension or diabetes in a systematic review (35). The incidence of CKD, rate of progression, and effectiveness of drug therapy were major drivers of cost-effectiveness and thus CKD screening may be more cost-effective in populations with higher incidences of CKD, rapid rates of progression, and more effective drug therapy.

## A rational approach to CKD early detection

The approach towards CKD early detection will include the decision for frequency of screening, who should perform the screening, and intervention after screening (21). Screening frequency for targeted individuals should be yearly if no abnormality is detected on initial evaluation. This is in line with the Kidney Disease Improving Global Outcomes (KDIGO) resolution that the frequency of testing should be according to the target group to be tested and generally needs not be more frequent than once per year (25). Who should perform the screening is always a question especially when the healthcare professional availability is a challenge in lower income countries. Physicians, nurses, paramedical staff, and other trained healthcare professionals are eligible to do the screening tests. Intervention after screening is also important and patients detected with CKD should be referred to primary care and general physicians with experience in management of kidney disease for follow up. A management protocol should be provided to the primary care and general physicians. Further referral to nephrologists for management should be based on well-defined protocols (22,25,27).

## Integration of CKD prevention into national NCD programs

Given the close links between CKD and other NCDs, it is critical that CKD advocacy efforts be aligned with existing initiatives concerning diabetes, hypertension, and cardiovascular disease, particularly in LMIC. Some countries and regions have successfully introduced CKD prevention strategies as part of their NCD programs. As an example, in 2003, a kidney health promotion program was introduced in Taiwan, with its key components including a ban on herbs containing aristolochic acid, public-awareness campaigns, patient education, funding for CKD research, and the setting up of teams to provide integrated care (36). In Cuba, the Ministry of Public Health has implemented a national program for the prevention of CKD. Since 1996 the program has followed several steps: 1) the analysis of the resources and health situation in the country; 2) epidemiological research to define the burden of CKD; and 3); continuing education for nephrologists, family doctors, and other health professionals. The main goal has been to bring nephrology care closer to the community through a regional redistribution of nephrology services and joint management of CKD patients by primary healthcare physicians and nephrologists (37). The integration of CKD prevention into NCDs program has resulted in the reduction of renal and cardiovascular risks in the general population. Main outcomes have been the reduction in the prevalence of risk factors, such as low birth weight, smoking, and infectious diseases. There has been an increased rate of the diagnosis of diabetes and of glycemic control, as well as an increased diagnosis of patients with hypertension, higher prescription use of renoprotective treatment with ACEI, and higher rates of blood pressure control (38). Recently, the US Department of Health and Human Services has introduced an ambitious program to reduce the number of Americans developing ESRD by 25 percent by 2030. The program, known as the Advancing American Kidney Health Initiative, has set goals with metrics to measure its success; among them is to increase efforts to prevent, detect, and slow the progression of kidney disease, in part by addressing traditional risk factors like diabetes and hypertension. To reduce the risk of kidney failure, the program contemplates advancing public health surveillance and research to identify populations at risk and those in early stages of kidney disease, and to encourage adoption of evidence-based interventions to delay or stop progression to kidney failure (39). Ongoing programs, like the Special Diabetes Program for Indians represents an important part of this approach by providing team-based care and care management. Since its implementation, the incidence of diabetes-related kidney failure among American Native populations decreased by over 40 percent between 2000 and 2015 (40).

## Involvement of primary care physicians and other health professionals

Detection and prevention of CKD programs require considerable resources both in terms of manpower and funds. Availability of such resources will depend primarily on the leadership of nephrologists (41). However, the number of nephrologists is not sufficient to provide renal care to the growing number of CKD patients worldwide. It has been suggested that most cases of non-progressive chronic kidney disease can be managed without referral to a nephrologist, and specialist referral can be reserved for patients with an estimated GFR rate <30 mL·min^-1^·(1.73 m^2^)^-1^, rapidly declining kidney function, persistent proteinuria, or uncontrolled hypertension or diabetes (42). It has been demonstrated that with an educational intervention the clinical competence of family physicians increases, resulting in preserved renal function in diabetic patients with early renal disease (43). The practitioners who received the educational intervention used significantly more angiotensin-converting enzyme inhibitors, angiotensin-receptor blockers, and statins than did practitioners who did not receive it. The results were similar to those found in patients treated by nephrologists (44). The role of primary health care professionals in the implementation of CKD prevention strategies in LMIC has been recently illustrated (45).

The e-learning has become an increasingly popular approach to medical education. Online learning programs for NCD prevention and treatment, including CKD, have been successfully implemented in Mexico. By 2015, over 5000 health professionals (including non-nephrologists) had been trained using an electronic health education platform (46).

## Shortage of nephrology manpower – implication on prevention

The resources for nephrology care remain at critical levels in many parts of the world. Even in Western developed countries, nephrologists are frequently in short supply. In a selection of European countries with similar, predominantly public, health care systems, there was a substantial variation in the nephrology workforce. Countries like Italy, Greece, and Spain reported the highest ratios, while countries like Ireland, Turkey, and the UK had the lowest ones (47). In the USA, the number of nephrologists per 1000 ESRD patients has declined over the years, from 18 in 1997 to 14 in 2010 (48). The situation in the developing world is even worse. With the exception of Nigeria, Sudan, Kenya, and South Africa, in many countries of sub-Saharan Africa there are fewer than 10 nephrologists. The number of nephrology nurses and dialysis technicians is also insufficient (49). In Latin America the average number of nephrologists is 13.4 pmp. However, there is unequal distribution between countries; some with <10 nephrologists pmp (Honduras, 2.1 pmp; Guatemala, 3.3 pmp; and Nicaragua, 4.6 pmp), and some exceeding 25 pmp (Cuba, 45.2 pmp; Uruguay, 44.2 pmp; and Argentina, 26.8 pmp) (50).

The causes of this shortage are multiple. Potential contributors to this variation include the increasing burden of CKD, erosion of nephrology practice scope by other specialists, lack of workforce planning in some countries relative to others, and the development of new care delivery models (48). A novel strategy has been the successful ISN Fellowship program. Since its implementation in 1985, over 600 fellows from >83 LMIC have been trained. A significant number of fellowships were undertaken in selected developed centers within the fellow's own region. In a recent survey, 85% of responding fellows were re-employed by their home institutions (51,52).

## Interdisciplinary prevention approach

Since 1994, a National Institutes of Health consensus advocated for early medical intervention in predialysis patients. Owing to the complexity of care of CKD, it was recommended that patients should be referred to a multidisciplinary team consisting of nephrologist, dietitian, nurse, social worker, and health psychologist with the aim to reduce predialysis and dialysis morbidity and mortality (53). In Mexico, a nurse-led, protocol driven, multidisciplinary program reported better preservation in eGFR and a trend of improvement of quality of care of CKD patients similar to those reported by other Multidisciplinary Clinic programs in the developed world. Additionally, more patients started dialysis non-emergently, and some obtained a pre-emptive kidney transplant. For those unable to obtain dialysis or who choose not to, a palliative care program is now being implemented (54). Care models supporting primary care providers or allied health workers achieved better effectiveness in slowing kidney function decline when compared to those providing specialty care. Future models should address region-specific causes of CKD, increase the quality of diagnostic capabilities, establish referral pathways, and provide better assessments of clinical effectiveness and cost-effectiveness (55).

## Online educational programs for CKD prevention and treatment

Whereas it is important to enhance the promotion and implementation of “Prevention” of kidney disease and kidney failure amongst healthcare professionals, it is equally important to promote “Prevention” with education programs for those at risk of kidney disease and kidney failure, and for the general population at large. It is a stepwise process, from awareness, engagement, participation, empowerment, and partnership. As highlighted above, in general, the health literacy of the general population is low. Awareness and understanding of kidney disease are inadequate. Education is key to engaging patients with kidney disease. It is the path to self-management and patient-centered care. Narva et al. found patient education is associated with better patient outcomes (56). Obstacles include the complex nature of kidney disease information, low baseline awareness, limited health literacy and numeracy, limited availability of CKD information, and lack of readiness to learn. New education approaches should be developed through research and quality improvement efforts. Schatell found web-based kidney education is helpful in supporting patient self-management (57).

The internet offers a wealth of resources on education. Understanding the types of internet sources that CKD patients use today can help renal professionals to point patients in the right direction. It is important that reputable healthcare organizations, preferably at a national level, facilitate easier access to health information on their websites (Supplementary Table S1). The mode of communication currently used by patients and the population at large is through the internet - websites, portals, and other social media, such as Facebook and Twitter. There are also free apps on popular mobile devices providing education on kidney disease. There is no shortage of information on the internet. The challenge is how to effectively “push” important healthcare information in a targeted manner, and to facilitate users seeking information in their efforts to “pull” relevant and reliable information from the internet. It is important the “pushing” of health information is targeted and specific, relevant for the condition (primary, secondary, or tertiary prevention), and is offered at the right time to the right recipient. It is possible with the use of information technology and informatics to provide relevant and targeted information for patients at high risk, coupling the information based on diagnosis and drugs prescribed. Engagement of professional society resources and patient groups is a crucial step to promote community partnership and patient empowerment on prevention. Additional resources may be available from charitable and philanthropic organizations.

## Renewed focus on prevention, raising awareness, and education

Given the pressing urgency pertaining to the need for increasing education and awareness on the importance of the preventive measures, we suggest the following goals to redirect the focus on plans and actions:

1. Empowerment through health literacy in order to develop and support national campaigns that bring public awareness to prevention of kidney disease.
2. Population-based approaches to manage key known risks for kidney disease, such as blood pressure control and effective management of obesity and diabetes.
3. Implementation of the World Health Organization ‘Best Buys' approach including screening of at-risk populations for CKD, universal access to essential diagnostics of early CKD, availability of affordable basic technologies and essential medicines, and task shifting from doctors to front-line healthcare workers to more effectively target progression of CKD and other secondary preventative approaches.

To that end, the motto ‘Kidney Health for Everyone, Everywhere' is more than a tagline or wishful thinking. It is an imperative policy that can be successfully achieved if policy makers, nephrologists, and healthcare professionals place prevention and primary care for kidney disease within the context of their Universal Health Coverage programs.

## Supplementary Material

Click here to view [pdf].
